# Fusion of Land-Based and Satellite-Based Localization Using Constrained Weighted Least Squares

**DOI:** 10.3390/s24082628

**Published:** 2024-04-20

**Authors:** Paihang Zhao, Linqiang Jiang, Tao Tang, Zhidong Wu, Ding Wang

**Affiliations:** Institute of Information Engineering, PLA Strategic Support Force Information Engineering University, Zhengzhou 450001, China; zhaopaihang@126.com (P.Z.); wang_ding814@aliyun.com (D.W.)

**Keywords:** fusion result, short-wave localization, satellite localization, Kalman filtering

## Abstract

Combining multiple devices for localization has important applications in the military field. This paper exploits the land-based short-wave platforms and satellites for fusion localization. The ionospheric reflection height error and satellite position errors have a great impact on the short-wave localization and satellite localization accuracy, respectively. In this paper, an iterative constrained weighted least squares (ICWLS) algorithm is proposed for these two kinds of errors. The algorithm converts the nonconvex equation constraints to linear constraints using the results of the previous iteration, thus ensuring convergence to the globally optimal solution. Simulation results show that the localization accuracy of the algorithm can reach the corresponding Constrained Cramér–Rao Lower Bound (CCRLB). Finally, the localization results of the two methods are fused using Kalman filtering. Simulations show that the fused localization accuracy is improved compared to the single-means localization.

## 1. Introduction

Source localization has important applications in wireless sensor networks, radar, navigation, and other fields [1,2,3,4]. Typical localization parameters include direction of arrival (DOA), time difference of arrival (TDOA), frequency difference of arrival (FDOA), and so on. Over-the-horizon (OTH) localization is very common for emitters such as aircrafts and warships in the military field. For important military targets, it is very necessary to utilize multiple means and multiple platforms to locate and surveillance them, and the positioning accuracy is higher compared to single means. For targets such as ships, the signals they send can usually be received by land-based shortwave stations as well as satellites. Therefore, it is necessary to study accurate and effective multi-platform localization algorithms [5].

For short-wave stations, the frequency range of the received signal is from 3 MHz to 30 MHz, and the signal usually arrives at the observatory station after reflection from the ionosphere. The state of the ionosphere has a great influence on the localization results, so it is necessary to use an appropriate ionosphere model. Recently, TDOA-based localization for short waves as a new method has attracted the interest of many scholars. A mathematical solution for short-wave TDOA-based localization was proposed in the study of Jain A [6]. Huang S [7] proposed a gradient-type algorithm based on the Quasi-Parabolic (QP) ionosphere model. Although the QP model can describe the ionospheric state more accurately and can give analytic equations for a ray path, it involves a number of ionospheric parameters, which brings more errors. Although the ionospheric virtual height (IVH) reflection model [8,9,10] is relatively simple, it contains the ionospheric reflection height as the main parameter, which can accurately reflect the state of the ionosphere and can effectively simplify the localization problem.

However, ionospheric reflection height errors can also have a significant impact on the localization results, so the influence on the localization algorithm needs to be considered. The time-varying property of the ionosphere leads to a large error in the estimation of the TDOA and thus low accuracy in TDOA-based localization for short-wave sources. In contrast, DOA-based localization for short-wave emitters has gained maturity, and the localization results are more stable. Wang [9] proposed a new algorithm for short-wave sources using orthogonal triangular decomposition (QR decomposition) based on the IVH model. The positioning accuracy of this algorithm can reach the corresponding Cramér–Rao Lower Bound (CRLB).

In addition, satellite-based localization is a high-precision method that typically utilizes the TDOA. A closed-form algorithm for TDOA-AOA hybrid localization was proposed in [11] based on weighted least squares (WLS). Ref. [12] analyzes the effect of earth constraints on multi-satellite localization and obtains the final result by weighting the positioning results under different constraints through weighting coefficients. However, the position of satellites is usually inaccurate due to orbital errors, altitude errors, and other reasons [13]. Therefore, satellite position errors need to be considered in order to improve the localization accuracy [14]. Ref. [15] corrects for satellite errors using the calibration source and proposed a Lagrange algorithm. Ref. [16] proposed a moving horizon estimation (MHE)-based technique to achieve source tracking using TDOA-FDOA measurements from multiple satellites.

The surveillance of enemy warships is an important application in the military field, which usually utilizes short waves or satellites for communication. Therefore, high-precision positioning is of great significance. However, short-wave positioning is greatly affected by ionospheric interference, while satellite positioning is affected by orbital errors. Combining multiple platforms to localize important sources can effectively improve the localization accuracy, which is a very effective means in practice. By combining land-based short-wave stations with satellites for localization, a hybrid localization algorithm based on a penalty function was proposed in [5]. But, Ref. [5] does not exploit the elevation information and does not consider the position errors of the satellite. Moreover, the equation constraints in the localization optimization problem are nonconvex, and thus the method can fall into a local optimal solution, which leads to a degradation of the localization accuracy.

In this paper, an iterative constrained weighted least squares (ICWLS) algorithm is proposed for sources such as warships transmitting multiple types of signals, which are localized using land-based short-wave stations and satellites. By introducing auxiliary variables, the DOA-based localization using short-wave stations and the TDOA-based localization using satellites can be modeled as optimization problems with two quadratic equation constraints, respectively. The quadratic nonconvex equation constraints are linearized using the results of the previous iteration [17,18], which converts the nonconvex constraints to linear constraints and ensures the algorithm can converge to the global optimal solution. Due to the difference in auxiliary variables between the DOA pseudo-linear equation and the TDOA pseudo-linear equation, a unified pseudo-linear equation cannot be established. Therefore, it is proposed in this paper that the localization results of the two methods are fused using Kalman filtering to improve the localization accuracy.

The mathematical symbols used in this paper and explanations are shown in Table 1. This paper is organized as follows: Section 2 introduces the localization scenario and the measurement model; Section 3 establishes the corresponding pseudo-linear equations; Section 4 derives the Constrained CRLB (CCRLB); Section 5 gives the proposed localization algorithms and the method for fusion; Section 6 shows the simulation results; and Section 7 is the conclusion.

## 2. Measurement Models

### 2.1. Localization Scenario

We assume a total of $N_{1}+N_{2}$ stations, including $N_{1}$ land-based stations and $N_{2}$ satellites. These stations are used to locate the source on the Earth’s surface. The longitude and latitude of the source are denoted as $\omega_{u,1}$ and $\omega_{u,2}$, respectively. The station is represented by $O_{1},O_{2},\cdots ,O_{N_{1}},O_{N_{1}+1},\cdots ,O_{N_{1}+N_{2}}$, where $O_{1},O_{2},\cdots ,O_{N_{1}}$ represents the land-based stations and $O_{N_{1}+1},\cdots ,O_{N_{1}+N_{2}}$ represents satellites. The longitude and latitude of the *j*th station are $\omega_{j,1}$ and $\omega_{j,2}$, $1\leq j\leq N_{1}+N_{2}$, respectively. There is a ground station for satellites, with a longitude and latitude of $\omega_{t,1}$ and $\omega_{t,2}$, respectively. Based on the Earth ellipsoid model, (1) provides the transformation relationship between the geodetic coordinate system ${\left[\omega_{1},\omega_{2},H\right]}^{T}$ ($\omega_{1}$ and $\omega_{2}$ denote the longitude and latitude, respectively, and *H* is the altitude) and the space rectangular coordinate system [19]:

(1) $$\left[\begin{array}{c} x\\ y\\ z \end{array}\right]=\left[\begin{array}{c} \left(\alpha +H\right)cos\left(\omega_{2}\right)cos\left(\omega_{1}\right)\\ \left(\alpha +H\right)cos\left(\omega_{2}\right)sin\left(\omega_{1}\right)\\ \left(\left(1-e^{2}\right)+H\right)sin\left(\omega_{2}\right) \end{array}\right]$$

$\alpha =R_{e}/\sqrt{1-{\left(esin(\omega_{1})\right)}^{2}}$, where $R_{e}$ = 6378.137 km is the radius of the Earth’s equator and $e=\sqrt{1-R_{p}^{2}/R_{e}^{2}}$ denotes the eccentricity. $R_{p}=6356.752$ km is the polar radius of the Earth. Therefore, based on (1), the position vector of the land-based station and satellite can be $s_{j}^{o}={\left[s_{j,x}^{o},s_{j,y}^{o},s_{j,z}^{o}\right]}^{T}$. The source position vector is represented as $u={\left[u_{x},u_{y},u_{z}\right]}^{T}$, and the ground station position vector is $t={\left[t_{x},t_{y},t_{z}\right]}^{T}$. Since the source is located on the Earth surface, u satisfies the following equation:

(2) $$u^{T}\Lambda_{1}u-R_{e}^{2}=0$$

where $\Lambda_{1}=diag\left\{1,1,1/(1-e^{2})\right\}$.

### 2.2. DOA Measurement Model for Land-Based Station

**Figure 1:**
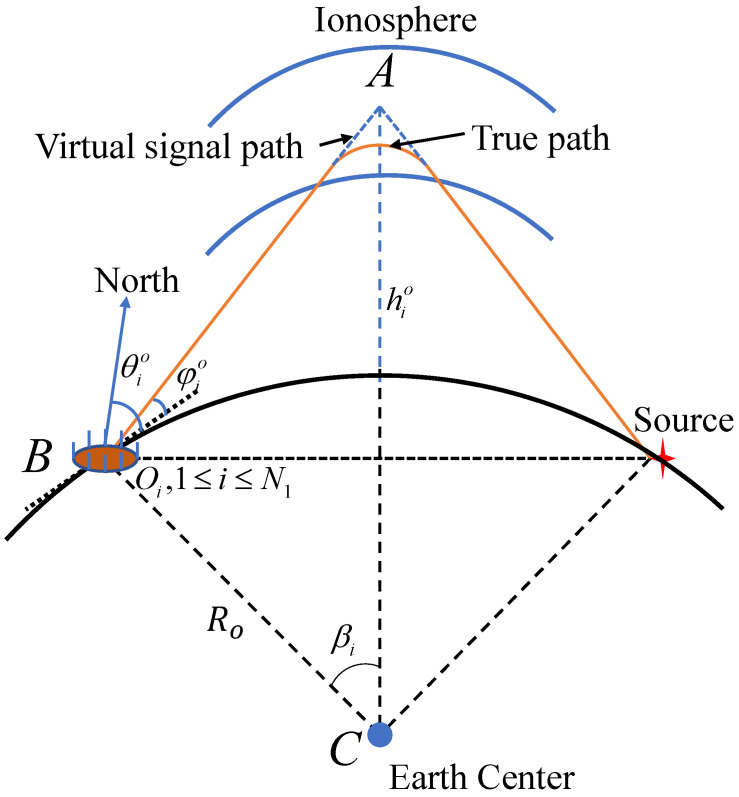
IVH model.

Figure 1 is the IVH model, which shows the short-wave signal sent by the source reaching the land-based station after being reflected by the ionosphere. $\theta_{i}^{o}$ represents the true value of the azimuth and $\varphi_{i}^{o}$ is the true value of elevation. $\theta_{i}^{o}$ is defined as the angle between the projection of the incidence direction on the surface plane and the local north direction of the station. $\varphi_{i}^{o}$ is defined as the angle between the incidence direction and the surface plane of the station. $h_{i}^{o}$ is the ionosphere reflection height and $R_{o}=6371.393$ km is the average radius of the Earth.

Combining all position vectors of land-based stations yields: $s_{a}^{o}=[{\left(s_{1}^{o}\right)}^{T},{\left(s_{2}^{o}\right)}^{T},\cdots ,$${\left(s_{N_{1}}^{o}\right)}^{T}{]}^{T}$. The ionosphere reflection height is often difficult to measure accurately and is therefore usually modeled as measurements with errors: $h_{i}=h_{i}^{o}+\delta h_{i}$, $1\leq i\leq N_{1}$. The reflection height measurement vector is thus given as $h={\left[h_{1},h_{2},\cdots ,h_{N_{1}}\right]}^{T}=h^{o}+n_{h}$, and $h^{o}={\left[h_{1}^{o},h_{2}^{o},\cdots ,h_{N_{1}}^{o}\right]}^{T}$ denotes the true value of the ionosphere reflection height. $n_{h}={\left[\delta h_{1},\delta h_{2},\cdots ,\delta h_{N_{1}}\right]}^{T}$ denotes the measurement error, and its covariance matrix is denoted as $Q_{h}$.

In the local coordinate system of the station, the target coordinate vector u can be transformed:

(3) $$u_{i}^{\prime }={\left[\begin{array}{ccc} -\sin(\omega_{i,1}) & \cos(\omega_{i,1}) & 0\\ -\cos\left(\omega_{i,1}\right)\sin\left(\omega_{i,2}\right) & -\sin\left(\omega_{i,1}\right)\sin\left(\omega_{i,2}\right) & \cos(\omega_{i,2})\\ \cos\left(\omega_{i,1}\right)\cos\left(\omega_{i,2}\right) & \sin\left(\omega_{i,1}\right)\cos\left(\omega_{i,2}\right) & \sin(\omega_{i,2}) \end{array}\right]}^{T}\left(u-s_{i}^{o}\right)$$

The origin of the local coordinate system is the station. Therefore, $\theta_{i}^{o}$ can be given as

(4) $$\theta_{i}^{o}=arctan\left(\frac{b_{i,1}^{T}\left(u-s_{i}^{o}\right)}{b_{i,2}^{T}\left(u-s_{i}^{o}\right)}\right)$$

where $b_{i,1}={\left[-sin\left(\omega_{i,1}\right),cos\left(\omega_{i,1}\right),0\right]}^{T}$, $b_{i,2}={\left[-cos\left(\omega_{i,1}\right)sin\left(\omega_{i,2}\right),-sin\left(\omega_{i,1}\right)sin\left(\omega_{i,2}\right),cos\left(\omega_{i,2}\right)\right]}^{T}$.

According to (4), the true value vector of azimuth can be given as $\theta^{o}={\left[\theta_{1}^{o},\theta_{2}^{o},\cdots ,\theta_{N_{1}}^{o}\right]}^{T}$. In practice, there will be measurement noise, so the following measurement vector can be obtained as:

(5) $$\theta ={\left[\theta_{1},\theta_{2},\cdots ,\theta_{N_{1}}\right]}^{T}=\theta^{o}+n_{\theta }\in R^{N_{1}\times 1}$$

where $n_{\theta }={\left[\delta \theta_{1},\delta \theta_{2},\ldots ,\delta \theta_{N_{1}}\right]}^{T}$ denotes the noise vector, which follows a zero-mean Gaussian distribution, and the covariance matrix is $Q_{\theta }=E\left(n_{\theta }n_{\theta }^{T}\right)$.

▵ABC, based on the sine theorem, yields

(6) $$\frac{h_{i}^{o}+R_{o}}{sin\left(\pi /2+\varphi_{i}^{o}\right)}=\frac{R_{o}}{sin\left(\pi -\left(\pi /2+\varphi_{i}^{o}+\beta_{i}\right)\right)}$$

where $\beta_{i}=arcsin\left(\frac{||u-s_{i}^{o}{\left|\right|}_{2}}{2R_{o}}\right)$. (6) can be converted into

(7) $$\varphi_{i}^{o}=arctan\left(\frac{\left(R_{o}+h_{i}^{o}\right)cos\left(\beta_{i}\right)-R_{o}}{\left(R_{o}+h_{i}^{o}\right)sin\left(\beta_{i}\right)}\right),1\leq i\leq N_{1}$$

Based on (7), the true value vector of the elevation angle is expressed as $\varphi^{o}={\left[\varphi_{1}^{o},\varphi_{2}^{o},\cdots ,\varphi_{N_{1}}^{o}\right]}^{T}$, and further, the measurement vector is given as

(8) $$\varphi ={\left[\varphi_{1},\varphi_{2},\cdots ,\varphi_{N_{1}}\right]}^{T}=\varphi^{o}+n_{\varphi }\in R^{N_{1}\times 1}$$

$n_{\varphi }={\left[\delta \varphi_{1},\delta \varphi_{2},\cdots ,\delta \varphi_{N_{1}}\right]}^{T}$ denotes the elevation noise vector, and the covariance matrix of $n_{\varphi }$ is $Q_{\varphi }$.

Combining (5) and (8) gives the DOA measurement model for land-based stations as

(9) $$z={\left[\theta^{T},\varphi^{T}\right]}^{T}=z^{o}+n_{a}$$

where $z^{o}={\left[{\left(\theta^{o}\right)}^{T},{\left(\varphi^{o}\right)}^{T}\right]}^{T}$ and $n_{a}={\left[n_{\theta }^{T},n_{\varphi }^{T}\right]}^{T}$. The covariance matrix of $n_{a}$ is $Q_{a}=blkdiag\left\{Q_{\theta },Q_{\varphi }\right\}$.

### 2.3. TDOA Measurement Model for Land-Based Station

**Figure 2:**
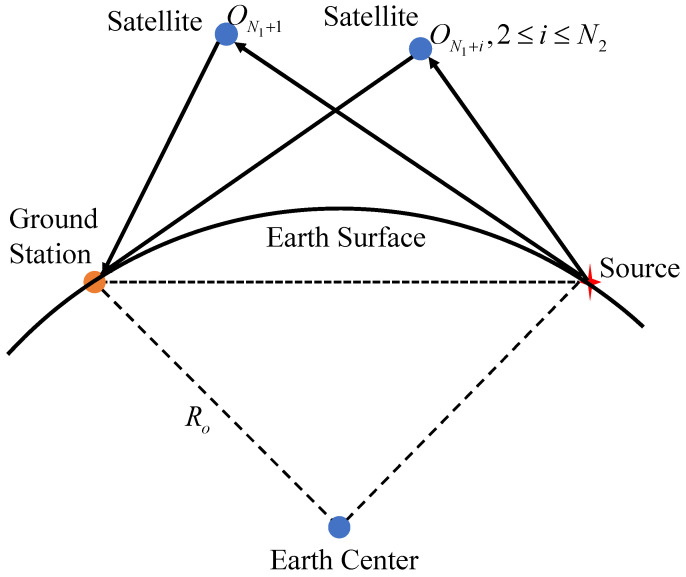
Schematic diagram of a satellite relaying the source signal.

The signals emitted by the source are forwarded by satellites to the ground station, which receives the signals sent by all the satellites and can obtain the TDOAs. Figure 2 shows the schematic diagram of the satellite forwarding the signal to the ground station.

Satellites are in high-speed motion and thus the positions are also in error, so the satellite positions are modeled as measurements with errors: $s_{N_{1}+j}=s_{N_{1}+j}^{o}+\delta s_{N_{1}+j}$, $1\leq j\leq N_{2}$, $\delta s_{N_{1}+j}={\left[\delta s_{N_{1}+j,x},\delta s_{N_{1}+j,y},\delta s_{N_{1}+j,z}\right]}^{T}$ is the random error. Thus, the satellite position vector is obtained as $s_{t}=s_{t}^{o}+n_{s}$, where $s_{t}^{o}={\left[{\left(s_{N_{1}+1}^{o}\right)}^{T},{\left(s_{N_{1}+2}^{o}\right)}^{T},...,{\left(s_{N_{1}+N_{2}}^{o}\right)}^{T}\right]}^{T}$. $n_{s}={\left[{\left(\delta s_{N_{1}+1}\right)}^{T}{\left(\delta s_{N_{1}+2}\right)}^{T},\ldots ,{\left(\delta s_{N_{1}+N_{2}}\right)}^{T}\right]}^{T}$ with the covariance matrix $Q_{s}$.

From Figure 2, the propagation length can be expressed as

(10) $$r_{j}^{o}=||u-s_{N_{1}+j}^{o}|{|}_{2}+\left|\right|s_{N_{1}+j}^{o}-t{\left|\right|}_{2},1\leq j\leq N_{2}$$

Taking the first satellite as the reference satellite, the TDOA can be obtained as

(11) $$\tau_{i1}^{o}=\frac{1}{c}\left(r_{i}^{o}-r_{1}^{o}\right),2\leq i\leq N_{2}$$

where *c* is the signal propagation velocity and is a known constant. Thus, the TDOA shown in (11) can be converted to the range difference of arrival (RDOA) shown in (12):

(12) $$\begin{array}{c} r_{i1}^{o}=||u-s_{N_{1}+i}^{o}|{|}_{2}-||u-s_{N_{1}+1}^{o}|{|}_{2}+\left|\right|s_{N_{1}+i}^{o}-t|{|}_{2}-\left|\right|s_{N_{1}+1}^{o}-t|{|}_{2},2\leq i\leq N_{2} \end{array}$$

The measurement vector of RDOA is denoted as

(13) $$r={\left[r_{21},r_{31},\ldots ,r_{N_{2}1}\right]}^{T}=r^{o}+n_{r}\in R^{(N_{2}-1)\times 1}$$

where $n_{r}={\left[\delta r_{21},\delta r_{31},\ldots ,\delta r_{N_{2}1}\right]}^{T}$ is the measurement noise with the covariance matrix $Q_{r}$.

## 3. Pseudo-Linear Equation

### 3.1. DOA Pseudo-Linear Equation for Land-Based Station

The linear equation of azimuth is first established. Based on (4), we have

(14) $$\sin\left(\theta_{i}^{o}\right)b_{i,2}^{T}\left(u-s_{i}^{o}\right)=\cos\left(\theta_{i}^{o}\right)b_{i,1}^{T}\left(u-s_{i}^{o}\right),1\leq i\leq N_{1}$$

Simplifying (14) gives

(15) $${\left(\sin\left(\theta_{i}^{o}\right)b_{i,2}-\cos\left(\theta_{i}^{o}\right)b_{i,1}\right)}^{T}u={\left(\sin\left(\theta_{i}^{o}\right)b_{i,2}-\cos\left(\theta_{i}^{o}\right)b_{i,1}\right)}^{T}s_{i}^{o},1\leq i\leq N_{1}$$

Combining (15) of all land-based stations obtains

(16) $$G_{\theta }^{o}u=y_{\theta }^{o}$$

where

(17) $$G_{\theta }^{o}=\left[\begin{array}{c} {\left(\sin\left(\theta_{1}^{o}\right)b_{1,2}-\cos\left(\theta_{1}^{o}\right)b_{1,1}\right)}^{T}\\ {\left(\sin\left(\theta_{2}^{o}\right)b_{2,2}-\cos\left(\theta_{2}^{o}\right)b_{2,1}\right)}^{T}\\ \vdots \\ {\left(\sin\left(\theta_{N_{1}}^{o}\right)b_{N_{1},2}-\cos\left(\theta_{N_{1}}^{o}\right)b_{N_{1},1}\right)}^{T} \end{array}\right]\in R^{N_{1}\times 3}$$

(18) $$y_{\theta }^{o}=\left[\begin{array}{c} {\left(\sin\left(\theta_{1}^{o}\right)b_{1,2}-\cos\left(\theta_{1}^{o}\right)b_{1,1}\right)}^{T}s_{1}^{o}\\ {\left(\sin\left(\theta_{2}^{o}\right)b_{2,2}-\cos\left(\theta_{2}^{o}\right)b_{2,1}\right)}^{T}s_{2}^{o}\\ \vdots \\ {\left(\sin\left(\theta_{N_{1}}^{o}\right)b_{N_{1},2}-\cos\left(\theta_{N_{1}}^{o}\right)b_{N_{1},1}\right)}^{T}s_{N_{1}}^{o} \end{array}\right]\in R^{N_{1}\times 1}$$

Converting (7) gives

(19) $$\left(R_{o}+h_{i}^{o}\right)\sin\left(\beta_{i}\right)\sin\left(\varphi_{i}^{o}\right)=\left(\left(R_{o}+h_{i}^{o}\right)\cos\left(\beta_{i}\right)-R_{o}\right)\cos\left(\varphi_{i}^{o}\right)$$

Substituting $\beta_{i}$ into (19) yields

(20) $$\left(R_{o}+h_{i}^{o}\right)\sin\left(\varphi_{i}^{o}\right)||u-s_{i}^{o}|{|}_{2}+2R_{o}^{2}\cos\left(\varphi_{i}^{o}\right)=\left(R_{o}+h_{i}^{o}\right)\cos\left(\varphi_{i}^{o}\right)\sqrt{4R_{o}^{2}-||u-s_{i}^{o}{\left|\right|}_{2}^{2}}$$

Squaring and simplifying both sides of (20) simultaneously yields

(21) $$c_{i,1}||u-s_{i}^{o}{\left|\right|}_{2}^{2}+c_{i,2}||u-s_{i}^{o}{\left|\right|}_{2}+c_{i,3}=0$$

where $c_{i,1}={\left(R_{o}+h_{i}^{o}\right)}^{2}$, $c_{i,2}=2R_{o}^{2}\left(R_{o}+h_{i}^{o}\right)\sin\left(2\varphi_{i}^{o}\right)$, and $c_{i,3}=-4R_{o}^{2}\left({\left(h_{i}^{o}\right)}^{2}+2R_{o}h_{i}^{o}\right){\left(\cos\left(\varphi_{i}^{o}\right)\right)}^{2}$.

(21) is a quadratic equation about $||u-s_{i}^{o}{\left|\right|}_{2}$. Appendix A proves that $||u-s_{i}^{o}{\left|\right|}_{2}$ is the only positive root of the equation shown in (21). Therefore, we have

(22) $$c_{i,4}=\frac{\sqrt{c_{i,2}^{2}-4c_{i,1}c_{i,3}}-c_{i,2}}{2c_{i,1}}=||u-s_{i}^{o}{\left|\right|}_{2}$$

Squaring both sides of (22) simultaneously yields

(23) $$2s_{i}^{oT}u-{\left||u|\right|}_{2}^{2}=\left|\right|s_{i}^{o}{\left|\right|}_{2}^{2}-c_{i,4}^{2},1\leq i\leq N_{1}$$

The pseudo-linear equation for elevation is obtained as follows:

(24) $$G_{\varphi }^{o}\eta_{a}^{o}=y_{\varphi }^{o}$$

where

(25) $$G_{\varphi }^{o}=\left[\begin{array}{cc} 2{\left(s_{1}^{o}\right)}^{T} & -1\\ 2{\left(s_{2}^{o}\right)}^{T} & -1\\ \vdots & \vdots \\ 2{\left(s_{N_{1}}^{o}\right)}^{T} & -1 \end{array}\right]\in R^{N_{1}\times 4},y_{\varphi }^{o}=\left[\begin{array}{c} \left|\right|s_{1}^{o}{\left|\right|}_{2}^{2}-c_{1,4}^{2}\\ \left|\right|s_{2}^{o}{\left|\right|}_{2}^{2}-c_{2,4}^{2}\\ \vdots \\ \left|\right|s_{N_{1}}^{o}{\left|\right|}_{2}^{2}-c_{N_{1}}^{2} \end{array}\right]\in R^{N_{1}\times 1}$$

(26) $$\eta_{a}^{o}=\left[\begin{array}{c} u\\ {\left||u|\right|}_{2}^{2} \end{array}\right]\in R^{4\times 1}$$

Combining (16) and (24) yields the DOA pseudo-linear equation as

(27) $$G_{a}^{o}\eta_{a}^{o}=y_{a}^{o}$$

where

(28) $$G_{a}^{o}=\left[\begin{array}{c} \begin{array}{cc} G_{\theta }^{o} & 0_{N_{1}\times 1} \end{array}\\ G_{\varphi }^{o} \end{array}\right]\in R^{2N_{1}\times 4},y_{a}^{o}=\left[\begin{array}{c} y_{\theta }^{o}\\ y_{\varphi }^{o} \end{array}\right]\in R^{2N_{1}\times 1},\eta_{a}^{o}=\left[\begin{array}{c} u\\ {\left||u|\right|}_{2}^{2} \end{array}\right]\in R^{4\times 1}$$

In (27), ${\left||u|\right|}_{2}^{2}$ is the auxiliary variable. $\eta_{a}^{o}$ satisfies the following equation:

(29) $${\left(\eta_{a}^{o}\right)}^{T}{\tilde{\Lambda }}_{2}\eta_{a}^{o}+\gamma_{1}^{T}\eta_{a}^{o}=0$$

where ${\tilde{\Lambda }}_{2}=diag\left\{1,1,1,0\right\}$ and $\gamma_{1}={\left[0,0,0,-1\right]}^{T}$.

### 3.2. TDOA Pseudo-Linear Equation for Satellite

Let $g_{i}^{o}=r_{i1}^{o}-\left|\right|s_{N_{1}+i}^{o}-t|{|}_{2}+\left|\right|s_{N_{1}+1}^{o}-t|{|}_{2}$. Shifting the terms and squaring both sides simultaneously of (10) yields

(30) $${\left(g_{i}^{o}+||u-s_{N_{1}+1}^{o}|{|}_{2}\right)}^{2}=||u-s_{N_{1}+i}^{o}{\left|\right|}_{2}^{2},2\leq i\leq N_{2}$$

Expanding and simplifying (30) yields

(31) $$2{\left(s_{N_{1}+i}^{o}-s_{N_{1}+1}^{o}\right)}^{T}u+2g_{i}^{o}\left|\right|u-s_{N_{1}+1}^{o}|{|}_{2}={\left(s_{N_{1}+i}^{o}\right)}^{T}s_{N_{1}+i}^{o}-{\left(s_{N_{1}+1}^{o}\right)}^{T}s_{N_{1}+1}^{o}-{\left(g_{i}^{o}\right)}^{2}$$

Therefore, the pseudo-linear equation for the satellite can be given as

(32) $$G_{t}^{o}\eta_{t}^{o}=h_{t}^{o}$$

where

(33) $$G_{t}^{o}=\left[\begin{array}{cc} 2{\left(s_{N_{1}+2}^{o}-s_{N_{1}+1}^{o}\right)}^{T} & 2g_{2}^{o}\\ 2{\left(s_{N_{1}+3}^{o}-s_{N_{1}+1}^{o}\right)}^{T} & 2g_{3}^{o}\\ \vdots & \vdots \\ 2{\left(s_{N_{1}+N_{2}}^{o}-s_{N_{1}+1}^{o}\right)}^{T} & 2g_{N_{2}}^{o} \end{array}\right]\in R^{(N_{2}-1)\times 4}$$

(34) $$h_{t}^{o}=\left[\begin{array}{c} s_{N_{1}+2}^{oT}s_{N_{1}+2}^{o}-s_{N_{1}+1}^{oT}s_{N_{1}+1}^{o}-{\left(g_{2}^{o}\right)}^{2}\\ s_{N_{1}+3}^{oT}s_{N_{1}+3}^{o}-s_{N_{1}+1}^{oT}s_{N_{1}+1}^{o}-{\left(g_{3}^{o}\right)}^{2}\\ \vdots \\ s_{N_{1}+N_{2}}^{oT}s_{N_{1}+N_{2}}^{o}-s_{N_{1}+1}^{oT}s_{N_{1}+1}^{o}-{\left(g_{N_{2}}^{o}\right)}^{2} \end{array}\right]\in R^{(N_{2}-1)\times 1}$$

(35) $$\eta_{t}^{o}=\left[\begin{array}{c} u\\ \left|\right|u-s_{N_{1}+1}^{o}|{|}_{2} \end{array}\right]\in R^{4\times 1}$$

In (35), $\left|\right|u-s_{N_{1}+1}^{o}|{|}_{2}$ is similarly an introduced auxiliary variable. And, $\eta_{t}^{o}$ satisfies

(36) $${\left(\eta_{t}^{o}\right)}^{T}{\tilde{\Lambda }}_{3}\eta_{t}^{o}+2\left[-{\left(s_{N_{1}+1}^{o}\right)}^{T},0\right]\eta_{t}^{o}=-{\left(s_{N_{1}+1}^{o}\right)}^{T}s_{N_{1}+1}^{o}$$

where ${\tilde{\Lambda }}_{3}=diag\left\{1,1,1,-1\right\}$.

**Remark** **1.** *Any two equations having the form (27) and (32) can be combined into one equation by expanding the matrix dimension. However, this is a simple stacking of matrices and vectors and does not take advantage of the connection between the two. Since the auxiliary variables utilized in (27) and (32) are different, the unknown variables in the two equations are also different. Thus, it is not possible to directly combine (27) and (32) into one equation. Although it is possible to combine (27) and (32) to obtain an equation, the combination of the two equations is a simple matrix combination that does not take advantage of the connection between the two equations.*

## 4. CCRLB

The CRLB gives the theoretical lower bound that unbiased estimation can achieve, and the CRLB can be given by the inverse of the Fisher information matrix (FIM).

### 4.1. CCRLB for Land-Based Station

Under the Gaussian noise assumption, the log-likelihood function of $z^{o}$ with respect to $\xi_{1}={\left[u^{T},{\left(h^{o}\right)}^{T}\right]}^{T}$ can be expressed as [20,21]

(37) $$\ln\left(p\left\{z^{o}|\xi_{1}\right\}\right)=\lambda_{a}-\frac{1}{2}{\left(z-z^{o}\right)}^{T}Q_{z}^{-1}\left(z-z^{o}\right)-\frac{1}{2}{\left(h-h^{o}\right)}^{T}Q_{h}^{-1}\left(h-h^{o}\right)$$

$\lambda_{a}$ is a constant. The corresponding FIM is denoted as

(38) $$\mathrm{FI}M_{DOA}=E\left(\frac{\partial \ln\left(p\left\{z^{o}|\xi_{1}\right\}\right)}{\partial \xi_{1}}\frac{\partial \ln\left(p\left\{z^{o}|\xi_{1}\right\}\right)}{\partial \xi_{1}^{T}}\right)=\left[\begin{array}{cc} F_{1}^{DOA} & F_{2}^{DOA}\\ {\left(F_{2}^{DOA}\right)}^{T} & F_{3}^{DOA} \end{array}\right]$$

where

(39) $$\left\{\begin{array}{c} F_{1}^{DOA}={\left(\partial z^{o}/\partial u^{T}\right)}^{T}Q_{z}^{-1}\left(\partial z^{o}/\partial u^{T}\right)\\ F_{2}^{DOA}={\left(\partial z^{o}/\partial u^{T}\right)}^{T}Q_{z}^{-1}\left(\partial z^{o}/\partial {\left(h^{o}\right)}^{T}\right)\\ F_{3}^{DOA}={\left(\partial z^{o}/\partial {\left(h^{o}\right)}^{T}\right)}^{T}Q_{z}^{-1}\left(\partial z^{o}/\partial {\left(h^{o}\right)}^{T}\right)+Q_{h}^{-1} \end{array}\right.$$

The CRLB for DOA-based localization can be given as

(40) $$\mathrm{CRL}B_{DOA}={\left(\mathrm{FI}M_{DOA}\right)}^{-1}=\left[\begin{array}{cc} X_{1}^{DOA} & X_{2}^{DOA}\\ {\left(X_{2}^{DOA}\right)}^{T} & X_{3}^{DOA} \end{array}\right]$$

where $X_{1}^{DOA}\in R^{3\times 3}$ gives the CRLB for source localization.

Since the source position vector satisfies (2), the CCRLB with the source constraint can be expressed as [9]

(41) $$\mathrm{CCRL}B_{DOA}=\mathrm{CRL}B_{DOA}-\mathrm{CRL}B_{DOA}\tilde{\Lambda }{\left({\tilde{\Lambda }}^{T}\mathrm{CRL}B_{DOA}\tilde{\Lambda }\right)}^{-1}{\tilde{\Lambda }}^{T}\mathrm{CRL}B_{DOA}$$

where $\tilde{\Lambda }={\left[\Lambda_{1}u\right]}^{T}$.

### 4.2. CCRLB for Satellite

In the presence of errors in the satellite position, the TDOA-based log-likelihood function can be expressed as

(42) $$\ln\left(p\left\{r^{o}|\xi_{2}\right\}\right)=\lambda_{t}-\frac{1}{2}{\left(r-r^{o}\right)}^{T}Q_{r}^{-1}\left(r-r^{o}\right)-\frac{1}{2}{\left(s_{t}-s_{t}^{o}\right)}^{T}Q_{s}^{-1}\left(s_{t}-s_{t}^{o}\right)$$

$\lambda_{t}$ is also a constant and $\xi_{2}={\left[u^{T},{\left(s_{t}^{o}\right)}^{T}\right]}^{T}$.

The FIM can be given as

(43) $$\mathrm{FI}M_{TDOA}=\left[\begin{array}{cc} F_{1}^{TDOA} & F_{2}^{TDOA}\\ {\left(F_{2}^{TDOA}\right)}^{T} & F_{3}^{TDOA} \end{array}\right]$$

where

(44) $$\left\{\begin{array}{c} F_{1}^{TDOA}={\left(\partial r^{o}/\partial u^{T}\right)}^{T}Q_{r}^{-1}\left(\partial r^{o}/\partial u^{T}\right)\\ F_{2}^{TDOA}={\left(\partial r^{o}/\partial u^{T}\right)}^{T}Q_{r}^{-1}\left(\partial r^{o}/\partial {\left(s_{t}^{o}\right)}^{T}\right)\\ F_{3}^{TDOA}={\left(\partial r^{o}/\partial {\left(s_{t}^{o}\right)}^{T}\right)}^{T}Q_{r}^{-1}\left(\partial r^{o}/\partial {\left(s_{t}^{o}\right)}^{T}\right)+Q_{s}^{-1} \end{array}\right.$$

The CRLB for TDOA-based localization is obtained as

(45) $$\mathrm{CRL}B_{TDOA}={\left(\mathrm{FI}M_{TDOA}\right)}^{-1}=\left[\begin{array}{cc} X_{1}^{TDOA} & X_{2}^{TDOA}\\ {\left(X_{2}^{TDOA}\right)}^{T} & X_{3}^{TDOA} \end{array}\right]$$

where $X_{1}^{TDOA}\in R^{3\times 3}$.

And, the CCRLB can be given as

(46) $$\mathrm{CCRL}B_{TDOA}=\mathrm{CRL}B_{TDOA}-\mathrm{CRL}B_{TDOA}\tilde{\Lambda }{\left({\tilde{\Lambda }}^{T}\mathrm{CRL}B_{TDOA}\tilde{\Lambda }\right)}^{-1}{\tilde{\Lambda }}^{T}\mathrm{CRL}B_{TDOA}$$

### 4.3. CCRLB for DOA-TDOA Hybrid Localization

Define the unknown vector $\xi ={\left[u^{T},{\left(h^{o}\right)}^{T},{\left(s_{t}^{o}\right)}^{T}\right]}^{T}$ and the measurement vector is $z=z^{o}+n$. $z^{o}={\left[{\left(\theta^{o}\right)}^{T},{\left(\varphi^{o}\right)}^{T},{\left(r^{o}\right)}^{T}\right]}^{T}$. The covariance matrix of n is $Q_{z}$. The FIM of hybrid localization can be given as

(47) $$\mathrm{FIM}=\left[\begin{array}{ccc} F_{1} & F_{2} & F_{3}\\ {\left(F_{2}\right)}^{T} & F_{4} & F_{5}\\ {\left(F_{3}\right)}^{T} & {\left(F_{5}\right)}^{T} & F_{6} \end{array}\right]$$

where

(48) $$\left\{\begin{array}{c} F_{1}={\left(\partial z^{o}/\partial u^{T}\right)}^{T}Q_{z}^{-1}\left(\partial z^{o}/\partial u^{T}\right)\\ F_{2}={\left(\partial z^{o}/\partial u^{T}\right)}^{T}Q_{z}^{-1}\left(\partial z^{o}/\partial {\left(h_{t}^{o}\right)}^{T}\right)\\ F_{3}={\left(\partial z^{o}/\partial {\left(u^{o}\right)}^{T}\right)}^{T}Q_{z}^{-1}\left(\partial z^{o}/\partial {\left(s_{t}^{o}\right)}^{T}\right)\\ F_{4}={\left(\partial z^{o}/\partial {\left(h^{o}\right)}^{T}\right)}^{T}Q_{z}^{-1}\left(\partial z^{o}/\partial {\left(h^{o}\right)}^{T}\right)+Q_{h}^{-1}\\ F_{5}={\left(\partial z^{o}/\partial {\left(h^{o}\right)}^{T}\right)}^{T}Q_{z}^{-1}\left(\partial z^{o}/\partial {\left(s_{t}^{o}\right)}^{T}\right)\\ F_{6}={\left(\partial z^{o}/\partial {\left(s_{o}^{t}\right)}^{T}\right)}^{T}Q_{z}^{-1}\left(\partial r^{o}/\partial {\left(s_{t}^{o}\right)}^{T}\right)+Q_{s}^{-1} \end{array}\right.$$

Therefore, the CRLB and CCRLB can be expressed as

(49) $$\mathrm{CRLB}={\mathrm{FIM}}^{-1}={\left[\begin{array}{ccc} F_{1} & F_{2} & F_{3}\\ {\left(F_{2}\right)}^{T} & F_{4} & F_{5}\\ {\left(F_{3}\right)}^{T} & {\left(F_{5}\right)}^{T} & F_{6} \end{array}\right]}^{-1}$$

(50) $$\mathrm{CCRLB}=\mathrm{CRLB}-\mathrm{CRLB}\bar{\Lambda }{\left({\bar{\Lambda }}^{T}\mathrm{CRL}B\bar{\Lambda }\right)}^{-1}{\bar{\Lambda }}^{T}\mathrm{CRLB}$$

where $\bar{\Lambda }=blkdiag\left(\tilde{\Lambda },0_{\left(N_{1}+3N_{2}\right)\times \left(N_{1}+3N_{2}\right)}\right)$.

## 5. Proposed Method

### 5.1. Proposed Method for Land-Based Station

#### 5.1.1. Localization Method for Land-Based Station

Since in the practical process, only the measurement with errors is available, the following error equation can be established:

(51) $$\varepsilon_{a}=y_{a}-G_{a}\eta_{a}$$

Substituting the measurements with errors into (25) gives $y_{a}$ and $G_{a}$. Performing first-order Taylor expansion for $y_{a}$ and $G_{a}$ yields

(52) $$\left\{\begin{array}{c} y_{a}\approx y_{a}^{o}+A_{1}n_{a}+A_{2}n_{h}\\ G_{a}\approx G_{a}^{o}+\sum_{d_{1}=1}^{2N_{1}}<z{>}_{d_{1}}B_{1,d_{1}}+\sum_{d_{2}=1}^{N_{1}}<h{>}_{d_{2}}B_{2,d_{2}} \end{array}\right.$$

where

(53) $$\left\{\begin{array}{c} A_{1}=\frac{\partial y_{a}}{\partial z^{T}},B_{1,d_{1}}=\frac{\partial G_{a}}{\partial <z{>}_{d_{1}}},1\leq d_{1}\leq 2N_{1}\\ A_{2}=\frac{\partial y_{a}}{\partial h^{T}}B_{2,d_{2}}=\frac{\partial G_{a}}{\partial <h{>}_{d_{2}}},1\leq d_{2}\leq N_{1} \end{array}\right.$$

Substituting (52) into (51) yields

(54) $$\begin{array}{cc} \; & C_{1}=\left(A_{1}-\left[B_{1,1}\eta_{a},B_{1,2}\eta_{a},...,B_{1,2N_{1}}\eta_{a}\right]\right)\in R^{2N_{1}\times 2N_{1}}\\ \; & C_{2}=\left(A_{2}-\left[B_{2,1}\eta_{a},B_{2,2}\eta_{a},...,B_{2,N_{1}}\eta_{a}\right]\right)\in R^{2N_{1}\times N_{1}} \end{array}$$

Therefore, the DOA localization problem for land-based stations can be modeled as

(55) $$\left\{\begin{array}{c} \min_{\eta_{a}}J_{a}(\eta_{a})={\left(y_{a}-G_{a}\eta_{a}\right)}^{T}W_{a}\left(y_{a}-G_{a}\eta_{a}\right)\\ s.t.\;u^{T}u-{\left||u|\right|}_{2}^{2}=0\\ \;u^{T}\Lambda_{1}u=R_{e}^{2}\; \end{array}\right.$$

$W_{a}$ is the weighted matrix, which is defined as

(56) $$W_{a}={\left(E[\varepsilon_{a}\varepsilon_{a}^{T}\;]\right)}^{-1}={\left(C_{1}Q_{a}C_{1}^{T}+C_{2}Q_{h}C_{2}^{T}\right)}^{-1}$$

As can be seen from (55), the cost function is convex and the equation constraints are nonconvex. The equation constraints are written in matrix form as

(57) $$D_{a}\eta_{a}=d_{a}$$

where

(58) $$D_{a}=\left[\begin{array}{cc} u^{T} & -1\\ u^{T}\Lambda_{1} & 0 \end{array}\right],d_{a}=\left[\begin{array}{c} 0\\ R_{e}^{2} \end{array}\right]$$

$D_{a}$ is related to u, so the estimate of u can be substituted into $D_{a}$ to obtain $D_{a}^{k}$ during the iteration process. $J_{a}(\eta_{a})$ in (55) can be expressed as

(59) $$J_{a}(\eta_{a})=y_{a}^{T}W_{a}y_{a}-2y_{a}^{T}W_{a}G_{a}\eta_{a}+\eta_{a}^{T}G_{a}^{T}W_{a}G_{a}\eta_{a}$$

$y_{a}^{T}W_{a}y_{a}$ is a constant with respect to $\eta_{a}$. Therefore, substituting the results of the *k*th iteration into (57), (55) can be converted to

(60) $$\left\{\begin{array}{c} \min_{\eta_{a}}\;J_{a}(\eta_{a})=\eta_{a}^{T}{\tilde{G}}_{a}\eta_{a}-2{\tilde{y}}_{a}^{T}\eta_{a}\\ s.t.\;D_{a}^{k}\eta_{a}=d_{a}^{k}\; \end{array}\right.$$

where ${\tilde{G}}_{a}=G_{a}^{T}W_{a}G_{a}$ and ${\tilde{y}}_{a}=G_{a}^{T}W_{a}y_{a}$. *k* is the iteration number. $D_{a}^{k}$ and $d_{a}^{k}$ can be obtained by substituting the *k*th iteration result $u_{a}^{k}$ into (58).

As can be seen in (60), the cost function is still convex. However, by using the results of the previous iteration, the nonconvex quadratic equation constraints can be converted to linear constraints, which can ensure that the algorithm converges to the globally optimal solution. Thereby, the optimal solution of (60) is [17,22]:

(61) $${\tilde{\eta }}_{a}^{k}={\left(D_{a}^{k}\right)}^{†}d_{a}^{k}+P_{a}^{k}\zeta_{a}^{k}$$

where $\zeta_{a}^{k}={\left(P_{a}^{k}{\tilde{G}}_{a}^{k}P_{a}^{k}\right)}^{†}P_{a}^{k}\left({\tilde{y}}_{a}^{k}-{\tilde{G}}_{a}^{k}{\left(D_{a}^{k}\right)}^{†}d_{a}^{k}\right)$ and $P_{a}^{k}=I-{\left(D_{a}^{k}\right)}^{†}D_{a}^{k}$.

Therefore, the iteration results can be updated by Equation (62):

(62) $${\hat{\eta }}_{a}^{k+1}=\kappa_{a}{\hat{\eta }}_{a}^{k}+\left(1-\kappa_{a}\right){\tilde{\eta }}_{a}^{k}$$

$0<\kappa_{a}<1$ denotes the weighting factor.

The initial estimation can usually be obtained by making $W_{a}=Q_{z}^{-1}$ and then obtaining an initial estimation based on ${\hat{\eta }}_{a}^{0}={\left(G_{a}^{T}W_{a}G_{a}\right)}^{-1}G_{a}^{T}W_{a}y_{a}$.

The DOA localization algorithm for land-based stations is summarized in Table 2.

**Table 2:** Summary of land-based localization.

**Step 1:** Set k=0 and choose appropriate $\delta_{a}>0$ and $\kappa_{a}$. Let $W_{a}=Q_{z}^{-1}$ to obtain the initial result;
**Step 2:** Set k=k+1. Substituting ${\hat{\eta }}_{a}^{k-1}$ and ${\hat{u}}_{a}^{k-1}$ into (56) and (58) yields $W_{a}^{k-1}$, $D_{a}^{k-1}$, and $d_{a}^{k-1}$;
**Step 3:** Calculate ${\tilde{\eta }}_{a}^{k}$ based on (61) and update the iteration result by (62);
**Step 4:** If
(63) $$\left|\right|{\hat{\eta }}_{a}^{k}-{\hat{\eta }}_{a}^{k-1}|{|}_{2}/\left|\right|{\hat{\eta }}_{a}^{k}|{|}_{2}<\delta_{a}$$ Stop the iteration and output ${\hat{\eta }}_{a}={\hat{\eta }}_{a}^{k}$. If not, go to **Step 2**.

#### 5.1.2. Covariance Matrix of DOA Result

The DOA localization error is defined as ${\hat{\eta }}_{a}$. Thus, $\Delta \eta_{a}={\hat{\eta }}_{a}-\eta_{a}^{o}$ is the optimal solution to the following problem:

(64) $$\left\{\begin{array}{c} \min_{\Delta \eta_{a}}{\left(G_{a}\Delta \eta_{a}-\varepsilon_{a}\right)}^{T}W_{a}\left(G_{a}\Delta \eta_{a}-\varepsilon_{a}\right)\\ s.t.\;{\left(\Delta \eta_{a}\right)}^{T}{\tilde{\Lambda }}_{1}\eta_{a}^{o}=0\\ \;\;{\left(\Delta \eta_{a}\right)}^{T}\left(2{\tilde{\Lambda }}_{2}\eta_{a}^{o}+\gamma_{1}\right)=0 \end{array}\right.$$

where ${\tilde{\Lambda }}_{1}=blkdiag\left(\Lambda_{1},0\right)$. The constraints in (64) are derived from (2) and (29), respectively.

$\Delta \eta_{a}$ can be represented as [5]

(65) $$\Delta \eta_{a}=\left(I_{4}-{\tilde{G}}_{a}^{-1}\Psi_{a}{\left(\Psi_{a}^{T}{\tilde{G}}_{a}^{-1}\Psi_{a}\right)}^{-1}\Psi_{a}^{T}\right){\tilde{G}}_{a}^{-1}G_{a}^{T}W_{a}\varepsilon_{a}$$

where $\Psi_{a}=\left[\psi_{a1},\psi_{a2}\right]=\left[{\tilde{\Lambda }}_{1}\eta_{a}^{o},2{\tilde{\Lambda }}_{2}\eta_{a}^{o}+\gamma_{1}\right]$.

The covariance matrix of $\Delta \eta_{a}$ is expressed as

(66) $$\mathrm{cov}\left(\Delta \eta_{a}\right)={\tilde{G}}_{a}^{-1/2}\left(\Pi^{⊥}\left[{\tilde{G}}_{a}^{-1/2}\Psi_{a}\right]\right){\tilde{G}}_{a}^{-1/2}$$

The covariance matrix of the source estimate can be obtained as

(67) $$\mathrm{cov}\left({\hat{u}}_{a}\right)=\left[I_{3},0_{3\times 1}\right]{\tilde{G}}_{a}^{-1/2}\left(\Pi^{⊥}\left[{\tilde{G}}_{a}^{-1/2}\Psi_{a}\right]\right){\tilde{G}}_{a}^{-1/2}{\left[I_{3},0_{3\times 1}\right]}^{T}$$

#### 5.1.3. Computational Complexity of Land-Based Localization

Table 3 gives the number of multiplications required for some elements.

### 5.2. Proposed Method for Satellite

#### 5.2.1. Localization Method for Satellite

Substituting the measurements into (32) gives

(68) $$\varepsilon_{t}=y_{t}-G_{t}\eta_{t}$$

Performing the first-order Taylor expansion of $y_{t}$ and $G_{t}$ gives

(69) $$\left\{\begin{array}{c} y_{t}\approx y_{t}^{o}+A_{3}n_{r}+A_{4}n_{s}\\ G_{t}\approx G_{t}^{o}+\sum_{d_{1}=1}^{N_{2}-1}<r{>}_{d_{1}}B_{3,d_{1}}+\sum_{d_{2}=1}^{3N_{2}}<s_{t}{>}_{d_{2}}B_{4,d_{2}} \end{array}\right.$$

where

(70) $$\left\{\begin{array}{c} A_{3}=\frac{\partial y_{t}}{\partial r^{T}},B_{3,d_{1}}=\frac{\partial G_{t}}{\partial <r{>}_{d_{1}}},1\leq d_{1}\leq N_{2}-1\\ A_{4}=\frac{\partial y_{t}}{\partial s_{t}^{T}}B_{4,d_{2}}=\frac{\partial G_{t}}{\partial <s_{t}{>}_{d_{2}}},1\leq d_{2}\leq 3N_{2} \end{array}\right.$$

Substituting (69) into (68) gives

(71) $$\begin{array}{cc} \; & C_{3}=\left(A_{3}-\left[B_{3,1}\eta_{t},B_{3,2}\eta_{t},...,B_{3,N_{1}-1}\eta_{t}\right]\right)\in R^{\left(N_{2}-1\right)\times \left(N_{2}-1\right)}\\ \; & C_{4}=\left(A_{4}-\left[B_{4,1}\eta_{t},B_{4,2}\eta_{t},...,B_{4,3N_{2}}\eta_{t}\right]\right)\in R^{\left(N_{2}-1\right)\times 3N_{2}} \end{array}$$

Therefore, the TDOA localization for the satellite can be modeled as

(72) $$\left\{\begin{array}{c} \min_{\eta_{t}}J_{t}\left(\eta_{t}\right)={\left(y_{t}-G_{t}\eta_{t}\right)}^{T}W_{t}\left(y_{t}-G_{t}\eta_{t}\right)\\ s.t.{\left(u-s_{N_{1}+1}^{o}\right)}^{T}u-{||u-s_{N_{1}+1}^{o}\left|\right|}_{2}^{2}={\left(u-s_{N_{1}+1}^{o}\right)}^{T}s_{N_{1}+1}^{o}\\ \;\;\;\;\;u^{T}\Lambda_{1}u=R_{e}^{2} \end{array}\right.$$

$W_{t}$ is defined as

(73) $$W_{t}={\left(C_{3}Q_{r}C_{3}^{T}+C_{4}Q_{s}C_{4}^{T}\right)}^{-1}$$

Similarly, to ensure that the algorithm converges to the global optimal, a transformation of the nonconvex equality constraint is required. Writing the two equality constraints in matrix form gives

(74) $$D_{t}\eta_{t}=d_{t}$$

where

(75) $$D_{t}=\left[\begin{array}{cc} {\left(u-s_{N_{1}+1}^{o}\right)}^{T} & ||u-s_{N_{1}+1}^{o}{\left|\right|}_{2}\\ u^{T}\Lambda_{1} & 0 \end{array}\right],d_{t}=\left[\begin{array}{c} {\left(u-s_{N_{1}+1}^{o}\right)}^{T}s_{N_{1}+1}^{o}\\ R_{e}^{2} \end{array}\right]$$

Substituting the results of the *d*th iteration into (72) gives

(76) $$\left\{\begin{array}{c} \min_{\eta_{t}}\;J_{t}\left(\eta_{t}\right)=\eta_{t}^{T}{\tilde{G}}_{t}\eta_{t}-2{\tilde{y}}_{t}^{T}\eta_{t}\\ s.t.\;D_{t}^{k}\eta_{t}=d_{t}^{k}\; \end{array}\right.$$

where ${\tilde{G}}_{t}=G_{t}^{T}W_{t}G_{t}$ and ${\tilde{y}}_{t}=G_{t}^{T}W_{t}y_{t}$.

The optimal solution of (76) is expressed as

(77) $${\tilde{\eta }}_{t}^{k}={\left(D_{t}^{k}\right)}^{†}d_{t}^{k}+P_{t}^{k}\zeta_{t}^{k}$$

where $\eta_{t}^{k}={\left(P_{t}^{k}{\tilde{G}}_{t}^{k}P_{t}^{k}\right)}^{†}P_{t}^{k}\left({\tilde{y}}_{t}^{k}-{\tilde{G}}_{t}^{k}{\left(D_{t}^{k}\right)}^{†}d_{t}^{k}\right)$ and $P_{t}^{k}=I-{\left(D_{t}^{k}\right)}^{†}D_{t}^{k}$.

The k+1th iteration result is obtained by the following equation:

(78) $${\hat{\eta }}_{t}^{k+1}=\kappa_{t}{\hat{\eta }}_{t}^{k}+\left(1-\kappa_{t}\right){\tilde{\eta }}_{t}^{k}$$

where $0\leq \kappa_{t}\leq 1$.

In summary, the TDOA localization algorithm for satellites is summarized in Table 4.

**Table 4:** Summary of satellite-based localization.

**Step 1:** Set k=0 and choose appropriate $\delta_{t}>0$ and $\kappa_{t}$. Let $W_{t}=Q_{r}^{-1}$ to obtain the initial result;
**Step 2:** Set k=k+1. Substituting ${\hat{\eta }}_{t}^{k-1}$ and ${\hat{u}}_{t}^{k-1}$ into (73) and (75) yields $W_{t}^{k-1}$, $D_{t}^{k-1}$, and $d_{t}^{k-1}$, respectively;
**Step 3:** Calculate ${\tilde{\eta }}_{t}^{k}$ based on (77) and update the iteration result by (78);
**Step 4:** If
(79) $$\left|\right|{\hat{\eta }}_{t}^{k}-{\hat{\eta }}_{t}^{k-1}|{|}_{2}/\left|\right|{\hat{\eta }}_{t}^{k}|{|}_{2}<\delta_{t}$$ Stop the iteration and output ${\hat{\eta }}_{t}={\hat{\eta }}_{t}^{k}$. If not, go to **Step 2**.

#### 5.2.2. Covariance Matrix of TDOA Result

Define the TDOA estimation error as $\Delta \eta_{t}={\hat{\eta }}_{t}-\eta_{t}^{o}$. Therefore, $\Delta \eta_{t}$ is the optimal solution to the following problem:

(80) $$\left\{\begin{array}{l} \min_{\Delta \eta_{t}}{\left(G_{t}\Delta \eta_{t}-\varepsilon_{t}\right)}^{T}W_{t}\left(G_{t}\Delta \eta_{t}-\varepsilon_{t}\right)\\ s.t.{\left(\Delta \eta_{t}\right)}^{T}{\tilde{\Lambda }}_{1}\eta_{t}^{o}=0\\ \;\;\;\;\;{\left(\Delta \eta_{t}\right)}^{T}\left(2{\tilde{\Lambda }}_{3}\eta_{t}^{o}+2\left[-{\left(s_{N_{1}+1}^{o}\right)}^{T},0\right]\right)=0 \end{array}\right.$$

$\Delta \eta_{t}$ is defined as

(81) $$\Delta \eta_{t}=\left(I_{4}-{\tilde{G}}_{t}^{-1}\Psi_{t}{\left(\Psi_{t}^{T}{\tilde{G}}_{t}^{-1}\Psi_{t}\right)}^{-1}\Psi_{t}^{T}\right){\tilde{G}}_{t}^{-1}G_{t}^{T}W_{t}\varepsilon_{t}$$

where $\Psi_{t}=\left[\psi_{t1},\psi_{t2}\right]=\left[{\tilde{\Lambda }}_{1}\eta_{t}^{o},2{\tilde{\Lambda }}_{3}\eta_{t}^{o}+2\left[-{\left(s_{N_{1}+1}^{o}\right)}^{T},0\right]\right]$.

The covariance matrix of $\Delta \eta_{t}$ can be given as

(82) $$\mathrm{cov}\left(\Delta \eta_{t}\right)={\tilde{G}}_{t}^{-1/2}\left(\Pi^{⊥}\left[{\tilde{G}}_{t}^{-1/2}\Psi_{t}\right]\right){\tilde{G}}_{t}^{-1/2}$$

Therefore, the covariance matrix of the source estimate is

(83) $$\mathrm{cov}\left({\hat{u}}_{t}\right)=\left[I_{3},0_{3\times 1}\right]{\tilde{G}}_{t}^{-1/2}\left(\Pi^{⊥}\left[{\tilde{G}}_{t}^{-1/2}\Psi_{t}\right]\right){\tilde{G}}_{t}^{-1/2}{\left[I_{3},0_{3\times 1}\right]}^{T}$$

#### 5.2.3. Computational Complexity of Satellite-Based Localization

Table 5 gives the number of multiplications required for some elements.

### 5.3. Localization Result Fusion

${\hat{u}}_{a}$ and ${\hat{u}}_{t}$ are asymptotically unbiased estimates which, respectively, obey the following distribution:

(84) $${\hat{u}}_{a}∼N\left(u,\mathrm{cov}\left({\hat{u}}_{a}\right)\right),{\hat{u}}_{t}∼N\left(u,\mathrm{cov}\left({\hat{u}}_{t}\right)\right)$$

Based on Kalman filtering [23], the DOA-based localization results and TDOA-based localization results can be fused to obtain

(85) $$\hat{u}={\hat{u}}_{a}+K\left({\hat{u}}_{a}-{\hat{u}}_{t}\right)$$

where *K* denotes the Kalman gain, which is defined as [24,25]

(86) $$K=\frac{trace\left(\mathrm{cov}\left({\hat{u}}_{a}\right)\right)}{trace\left(\mathrm{cov}\left({\hat{u}}_{a}\right)\right)+trace\left(\mathrm{cov}\left({\hat{u}}_{t}\right)\right)}$$

**Figure 3:**
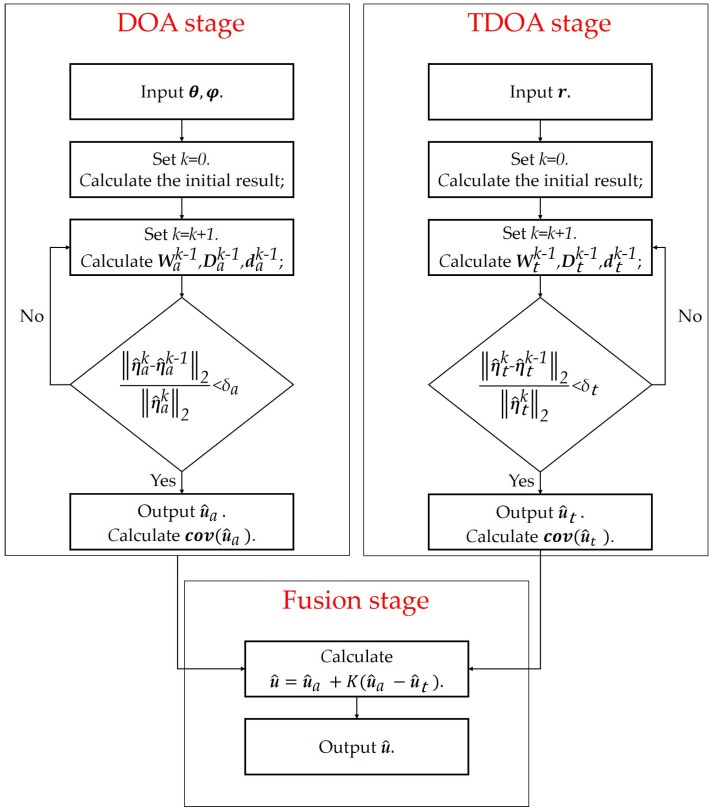
Block diagram for the proposed method.

The details of the proposed method are given in Figure 3. The computational complexity of the proposed method can be obtained by combining Table 3 and Table 5, although the proposed method has an increased computational complexity compared to land-based localization or satellite-based localization. However, since $N_{1}$ and $N_{2}$ are both small, the increase in computation is acceptable.

## 6. Simulation

Simulations are set up with a total of 10 observation stations, including 5 land-based stations and 5 satellites. The longitude and latitude of the land-based station and the corresponding ionospheric reflection heights are shown in Table 6. The longitude and latitude of the satellite and its altitude are shown in Table 7. The ground station is located at $\left(110.3^{\circ }\;E,\;25.7^{\circ }\;N\right)$. The source longitude and latitude are $\left(126^{\circ }\;E,\;37^{\circ }\;N\right)$, respectively.

**Remark** **2.** *The main application scenario of this paper is the military field, and the targets are mainly military targets such as warships. In addition, limited to the current level of the authors, no publicly available short-wave dataset has been found. The method proposed in this paper can not be tested on public datasets. Therefore, this paper uses simulation to verify the performance of the proposed method.*

Assuming that the noise between each parameter is uncorrelated, the covariance matrix can be set as $Q_{\theta }=\sigma_{\theta }^{2}I_{N_{1}}$, $Q_{\varphi }=\sigma_{\varphi }^{2}I_{N_{1}}$, $Q_{h}=\sigma_{h}^{2}I_{N_{1}}$, $Q_{s}=\sigma_{s}^{2}I_{3N_{2}}$, $Q_{r}=\sigma_{r}^{2}R_{N_{2}-1}$, where $R_{N_{2}-1}$ denotes the $\left(N_{2}-1\right)\times \left(N_{2}-1\right)$ matrix with a diagonal element of 1 and the rest of the elements are 0.5.

The root mean square error (RMSE) is used as the localization accuracy criterion and is defined as follows:

(87) $$RMSE\left({\hat{u}}_{a}\right)=\sqrt{\frac{\sum_{j=1}^{M}\left|\right|{\hat{u}}_{a}-u{\left|\right|}_{2}^{2}}{M}},RMSE\left({\hat{u}}_{t}\right)=\sqrt{\frac{\sum_{j=1}^{M}\left|\right|{\hat{u}}_{t}-u{\left|\right|}_{2}^{2}}{M}}$$

where *M* denotes the number of Monte Carlo experiments. Set $\delta_{a}=\delta_{t}=10^{-6}$, $\kappa_{a}=0.9^{k}$, and $\kappa_{t}=0.9^{k}$, where *k* denotes the number of iterations. The maximum number of iterations is set to 50.

### 6.1. Error Ellipse

**Figure 4:**
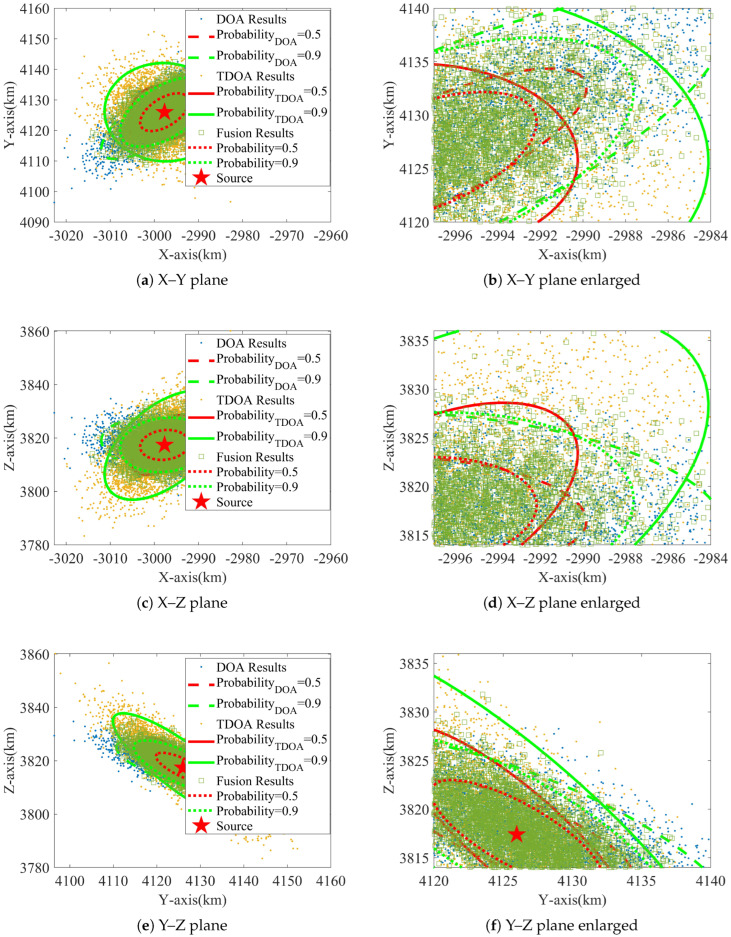
Localization results and uncertainty error ellipses.

Set $\sigma_{\theta }=0.5^{\circ }$, $\sigma_{\varphi }=0.75^{\circ }$, $\sigma_{h}=2$ km, $\sigma_{r}=0.5$ km, and $\sigma_{s}=1.5$ km. The localization results and the corresponding uncertainty error ellipses are given in Figure 4, with ellipse probabilities of 0.5 and 0.9, respectively.

As can be seen from Figure 4, the localization results are consistent with the ellipse, proving the effectiveness of the proposed ICWLS algorithm. In addition, the ellipse area of the fusion result is smaller compared to the single-method localization, which demonstrates that the fusion method improves the localization accuracy.

### 6.2. Localization Accuracy

This subsection simulates the localization accuracy of the proposed algorithm under the influence of different errors. Set $\sigma_{\theta }=0.2\sigma_{1}$, $\sigma_{\varphi }=0.3\sigma_{1}$, and $\sigma_{r}=0.3\sigma_{1}$, where $\sigma_{1}$ is the localization parameter error. Let $\sigma_{h}=0.5\sigma_{2}$ and $\sigma_{s}=0.2\sigma_{2}$, where $\sigma_{2}$ is the systematic error parameter.

**Remark** **3.** *The effects of the signal-noise ratio (SNR) and propagation channel will ultimately affect the estimation accuracy of the DOA and TDOA. Therefore, the error parameters $\sigma_{1}$ and $\sigma_{2}$ can ultimately reflect the individual errors. This paper focuses on the localization algorithm and thus mainly simulates the effect of the DOA error and TDOA error on the positioning accuracy.*

**Figure 5:**
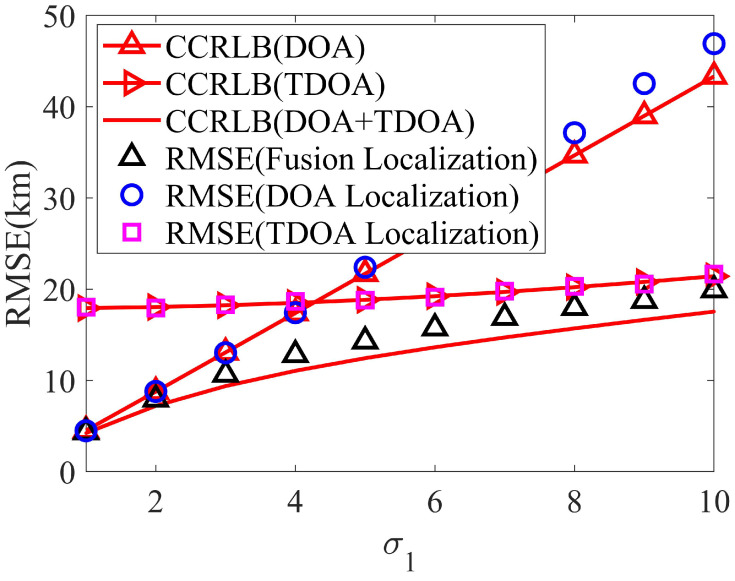
The localization accuracy varies with localization parameter error.

**Figure 6:**
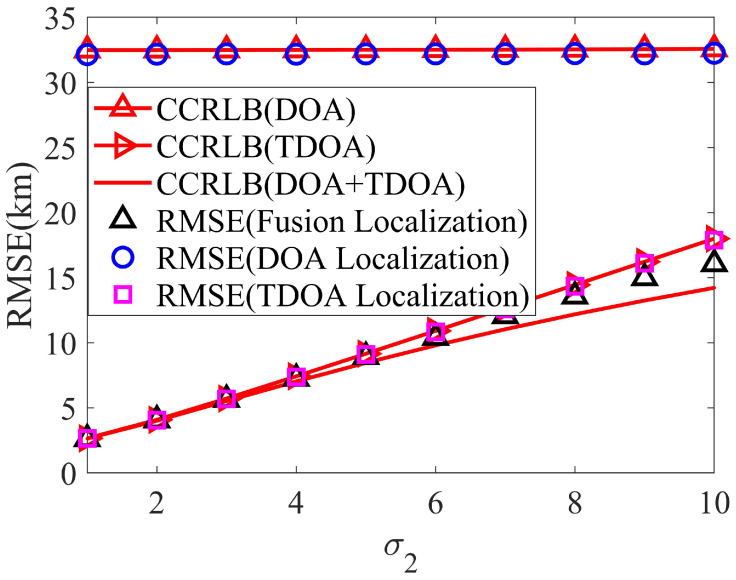
The localization accuracy varies with systematic error.

Firstly, set $\sigma_{h}=3$ km and $\sigma_{s}=2$ km and simulate the localization accuracy with $\sigma_{1}$. Figure 5 is the localization result. Then, let $\sigma_{\theta }=1.5^{\circ }$, $\sigma_{\varphi }=2.25^{\circ }$, and $\sigma_{r}=0.5$ km, and Figure 6 is the corresponding result.

From Figure 5 and Figure 6, the positioning accuracy of the proposed ICWLS algorithm for land-based DOA localization and satellite TDOA localization can reach the corresponding CCRLB. In addition, it can be seen that the fusion result can significantly improve the localization accuracy, which further proves the effectiveness of the proposed fusion method.

### 6.3. Robustness

A total of 10 sources are randomly selected within the range of $[120^{\circ }\;E$∼$130^{\circ }\;E]\times [20^{\circ }\;N$∼$30^{\circ }\;N]$. The localization accuracy of the proposed algorithm and the corresponding CCRLB distribution are tabulated.

**Figure 7:**
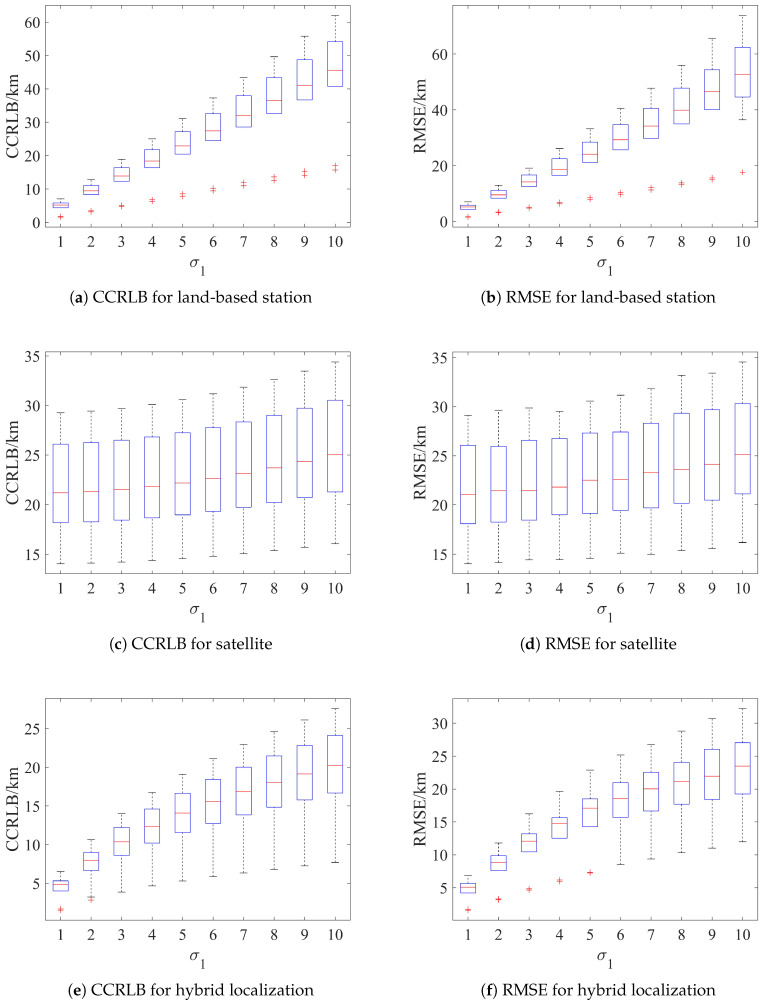
Distribution of RMSE and CCRLB with localization parameter error. `+’ is the outlier.

**Figure 8:**
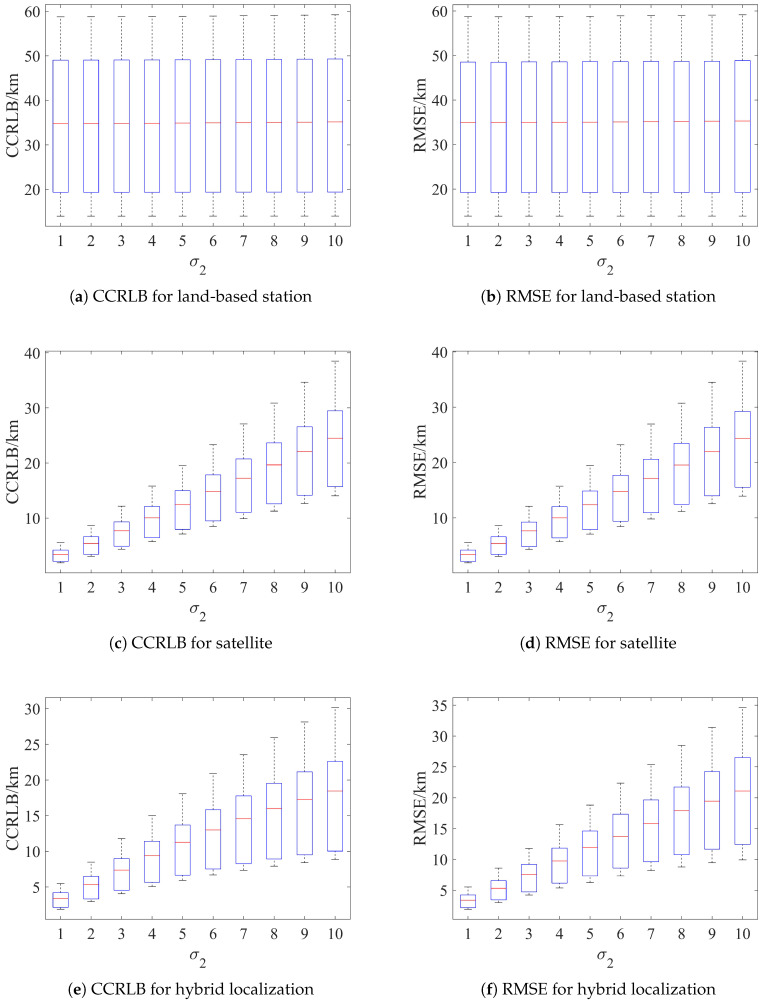
Distribution of RMSE and CCRLB with systematic error.

First, set $\sigma_{h}=3$ km and $\sigma_{s}=2$ km, and the corresponding results are shown in Figure 7. Then, set $\sigma_{\theta }=1.5^{\circ }$, $\sigma_{\varphi }=2.25^{\circ }$, and $\sigma_{r}=0.5$ km, and the results are shown in Figure 8.

As can be seen from Figure 7 and Figure 8, both the RMSE and CCRLB increase as the error increases, and the distribution is more dispersed. In addition, the distribution of the RMSE is basically the same as that of the CCRLB, and the localization results do not produce abnormal distribution values, which indicates that the proposed algorithm has good robustness in terms of the source location.

## 7. Conclusions

This paper investigated the fusion localization of land-based stations and satellites. And, the corresponding ICWLS algorithm is proposed for DOA-based localization and TDOA-based localization, respectively. Firstly, the pseudo-linear equations are established by using auxiliary variables, and then the localization problem is modeled as an optimization problem with two quadratic equation constraints. To ensure that the algorithm can converge to the global optimal solution, the results of the previous iteration are substituted into the equation constraints so that the nonconvex quadratic constraints can be converted into linear constraints. Simulations show that the localization accuracy of the proposed method can reach the corresponding CCRLB. In addition, this paper proposed to fuse the DOA-based localization and TDOA-based localization results using Kalman filtering. The simulation results show that the accuracy of fusion result is higher than the DOA-based localization and TDOA-based localization.

Although the proposed method can improve the positioning accuracy, the method requires the ability to receive signals from different frequencies. In addition, the method does not take into account the errors caused by the ionospheric multi-path effect and other effects. These will be studied in depth in the next step. In addition, we will further consider the practical application of the proposed method.

## Figures and Tables

**Table 1:** Mathematical symbols and explanations.

Symbols	Explanations
${\left[\cdot \right]}^{T}$	Matrix or vector transpose
${\left[\cdot \right]}^{†}$	Generalized inverses
${\left[\cdot \right]}^{-1}$	The inverse of the matrix
trace(·)	Traces of the matrix
E[·]	Mathematical expectation
$diag\left\{\cdot \right\}$	Diagonal matrix
$blkdiag\left\{\cdot \right\}$	Chunked diagonal matrix
$\left|\right|\cdot {\left|\right|}_{2}$	Euclidean norm
$<\cdot {>}_{i}$	The *i*th element of the vector
$\prod^{⊥}\left[A\right]$	Orthogonal projection matrix onto the orthogonal subspace of range A
$R^{m\times n}$	Real matrices of size m×n

**Table 3:** Computational complexity of land-based localization.

Computational Element	Computational Complexity
$G_{a}^{o}$	3$N_{1}$ + 4$N_{1}$
$y_{a}^{o}$	$2N_{1}$
${\hat{\eta }}_{a}^{0}$	$8N_{1}^{2}+32N_{1}+O\left(4^{3}\right)$
$W_{a}^{k}$ and ${\left(W_{a}^{k}\right)}^{-1}$	$2{\left(2N_{1}\right)}^{3}+6N_{1}^{3}+O\left({\left(2N_{1}\right)}^{3}\right)$
${\tilde{G}}_{a}^{k}$	$16N_{1}^{2}+32N_{1}$
${\tilde{y}}_{a}^{k}$	$8{\left(2N_{1}\right)}^{2}$
$P_{a}^{k}$	$32+O\left(2^{3}\right)$
$\zeta_{a}^{k}$	$280+O\left(2^{3}\right)+O\left(4^{3}\right)$
${\hat{\eta }}_{a}^{k}$	$56+O\left(2^{3}\right)$
$\mathrm{cov}\left({\hat{u}}_{a}\right)$	$276+O\left(2^{3}\right)$

**Table 5:** Computational complexity of satellite-based localization.

Computational Element	Computational Complexity
$G_{t}^{o}$	$4\left(N_{2}-1\right)$
$y_{t}^{o}$	$N_{2}-1$
${\hat{\eta }}_{t}^{0}$	$8{\left(N_{2}-1\right)}^{2}+32\left(N_{2}-1\right)+O\left(4^{3}\right)$
$W_{t}^{k}$ and ${\left(W_{t}^{k}\right)}^{-1}$	$\left(3N_{2}+2\right){\left(N_{2}-1\right)}^{2}+{\left(3N_{2}\right)}^{2}\left(N_{2}-1\right)+O\left({\left(N_{2}-1\right)}^{3}\right)$
${\tilde{G}}_{t}^{k}$	$4{\left(N_{2}-1\right)}^{2}+16\left(N_{2}-1\right)$
${\tilde{y}}_{t}^{k}$	$4{\left(N_{2}-1\right)}^{2}+4\left(N_{2}-1\right)$
$P_{t}^{k}$	$32+O\left(2^{3}\right)$
$\eta_{t}^{k}$	$280+O\left(2^{3}\right)+O\left(4^{3}\right)$
${\hat{\eta }}_{t}^{k}$	$56+O\left(2^{3}\right)$
$\mathrm{cov}\left({\hat{u}}_{t}\right)$	$276+O\left(2^{3}\right)$

**Table 6:** Longitude and latitude $\left(E:\;\mathrm{east},\;N:\;\mathrm{north}\right)$ and the corresponding ionospheric reflection height for land-based stations.

Station	1	2	3	4	5
**Longitude**	$119.27^{\circ }\;E$	$114.03^{\circ }\;E$	$114.54^{\circ }\;E$	$112.54^{\circ }\;E$	$116.00^{\circ }\;E$
**Latitude**	$26.05^{\circ }\;N$	$30.58^{\circ }\;N$	$38.04^{\circ }\;N$	$33.00^{\circ }\;N$	$29.71^{\circ }\;N$
**Reflection height**	365.00	385.00	350.00	390.00	370.00

**Table 7:** Longitude and latitude $\left(E:\;\mathrm{east},\;N:\;\mathrm{north}\right)$ and the corresponding altitude for satellites.

Satellite	1	2	3	4	5
**Longitude**	$124.32^{\circ }\;E$	$118.35^{\circ }\;E$	$120.87^{\circ }\;E$	$116.56^{\circ }\;E$	$122.54^{\circ }\;E$
**Latitude**	$30.57^{\circ }\;N$	$29.19^{\circ }\;N$	$32.65^{\circ }\;N$	$27.32^{\circ }\;N$	$29.62^{\circ }\;N$
**Altitude (km)**	1000.00	1000.00	1200.00	1200.00	1200.00

## Data Availability

Dataset available on request from the authors.

## Appendix A

In a practical scenario, $R_{o}$ and $h_{i}^{o}$ are both positive. In addition, the range of elevation is $0<\varphi_{i}^{o}<\pi /2\;$, so $\cos(\varphi_{i}^{o})>0$ and $\sin\left(2\varphi_{i}^{o}\right)>0$ are valid. Based on the definition of $c_{i,1}$, $c_{i,2}$, and $c_{i,3}$, we have $c_{i,1}>0$, $c_{i,2}>0$, $c_{i,3}<0$. Therefore, the discriminant of (21) is

(A1) $${\left(c_{i,2}\right)}^{2}-4c_{i,1}c_{i,3}>0$$

Thus, the two solutions of (21) can be given as

(A2) $$x_{1}=\frac{-c_{i,2}+\sqrt{c_{i,2}^{2}-4c_{i,1}c_{i,3}}}{2c_{i,1}},x_{2}=\frac{-c_{i,2}-\sqrt{c_{i,2}^{2}-4c_{i,1}c_{i,3}}}{2c_{i,1}}$$

Therefore, $x_{1}>0$ and $x_{2}<0$ are valid. $x_{1}>0$ is the only positive solution of (21).
